# Urgent endoscopic retrograde cholangiopancreatography is not superior to early ERCP in acute biliary pancreatitis with biliary obstruction without cholangitis

**DOI:** 10.1371/journal.pone.0190835

**Published:** 2018-02-05

**Authors:** Hee Seung Lee, Moon Jae Chung, Jeong Youp Park, Seungmin Bang, Seung Woo Park, Si Young Song, Jae Bock Chung

**Affiliations:** Department of Internal Medicine, Institute of Gastroenterology, Yonsei University College of Medicine, Seoul, Korea; University Hospital Llandough, UNITED KINGDOM

## Abstract

Acute pancreatitis is a common diagnosis worldwide, with gallstone disease being the most prevalent cause (50%). The American College of Gastroenterology recommends urgent endoscopic retrograde cholangiopancreatography (ERCP) (within 24 h) for patients with biliary pancreatitis accompanied by cholangitis. Most international guidelines recommend that ERCP be performed within 72 h in patients with biliary pancreatitis and a bile duct obstruction without cholangitis, but the optimal timing for endoscopy is controversial. We investigated the optimal timing for ERCP in patients with biliary pancreatitis and a bile duct obstruction without cholangitis, and whether performing endoscopy within 24 h is superior to performing it after 24 h. We analyzed the clinical data of 505 patients with newly diagnosed acute pancreatitis, from January 1, 2005 to December 31, 2014. We divided the patients into two groups according to the timing of ERCP: < 24 h (urgent) and 24–72 h (early).Among the 505 patients, 73 were diagnosed with biliary pancreatitis and a bile duct obstruction without cholangitis. The mean age of the patients was 55 years (range: 26–90 years). Bile duct stones and biliary sludge were identified on endoscopy in 45 (61.6%) and 11 (15.0%) patients, respectively. The timing of ERCP within 72 h was not associated with ERCP-related complications (*P* = 0.113), and the total length of hospital stay was not different between urgent and early ERCP (5.9 vs. 5.7 days, *P =* 0.174). No significant differences were found in total length of hospitalization or procedural-related complications, in patients with biliary pancreatitis and a bile duct obstruction without cholangitis, according to the timing of ERCP (< 24 h vs. 24–72 h).

## Introduction

Acute biliary pancreatitis (ABP), which is the most common form of pancreatitis, develops as a result of transient obstruction of the bile duct and pancreatic duct, which results in bile reflux or increased hydrostatic pressure in the pancreatic duct [1]. Endoscopists who perform endoscopic retrograde cholangiopancreatography (ERCP) frequently perform urgent ERCP in patients with ABP and a concomitant biliary obstruction, in the belief that this procedure reduces morbidity and mortality.

However, most cases of ABP are self-limiting and improve with conservative treatment; this is because most gallstones that cause ABP spontaneously pass to the duodenum. Moreover, the incidence of post-ERCP complications, including pancreatitis, bleeding, cholangitis, bowel perforation, and cholecystitis is relatively high [2]. Cavdar et al. [3] reported that up to 15% of stones seen during an acute attack of ABP pass spontaneously after the attack. However, in a few patients, persistent bile duct stones can lead to ongoing pancreatic duct or bile duct obstructions, in turn leading to severe acute pancreatitis or cholangitis. Therefore, urgent ERCP (within 24 h) after admission is recommended in patients with cholangitis, and early ERCP (within 72 h) after admission is recommended in patients with evidence of a biliary obstruction without cholangitis [4]. However, there is no definite consensus concerning the optimal timing of ERCP in such patients.

Despite the many studies on ERCP in patients with ABP, and the availability of clinical practice guidelines, it remains controversial whether endoscopists should perform ERCP in patients with ABP and biliary obstruction within 24 h [2,5,6]. We retrospectively reviewed patients with ABP and a bile duct obstruction without cholangitis to evaluate the optimal timing for ERCP in these patients.

## Materials and methods

### Patients

We retrospectively collected clinical data from patients who had been diagnosed with acute pancreatitis at Severance Hospital, South Korea from January 2005 to December 2014. Among the patients, we excluded those with the following concomitant conditions or characteristics: 1) cholangitis, which was defined as total bilirubin > 1.2 mg/dL and body temperature > 38.4°C [7], 2) age < 20 or > 90 years, 3) known bleeding disorder or severe coagulopathy that could not be sufficiently corrected, such as decompensated liver cirrhosis or idiopathic thrombocytopenia (platelet count < 50,000 cells/ml), 4) prolonged international normalized ratio of prothrombin time > 1.5, 5) time to ERCP > 72 h after admission, and 6) cholecystectomy during admission (because simultaneous cholecystectomy prolongs the total length of hospitalization; S1 Table). ERCP was done during the daytime on weekdays because the endoscopic room was open and ERCP was readily available. In addition, ERCP was not performed emergently on weekends if the patient was not indicated for urgent ERCP. Therefore, it was possible to collect a relatively similar amount of data according to ERCP timing after admission. We divided the patients with biliary pancreatitis into two groups: those receiving ERCP in < 24 h after admission (urgent ERCP) and those receiving ERCP 24–72 h after admission (early ERCP). All initial laboratory analyses were obtained within 24 h of admission, and imaging studies were performed within the first 48 h of admission. This study was performed in accordance with the ethical guidelines of the 1975 Declaration of Helsinki, and approved waa granted by the Institutional Review Board of Severance Hospital (approval number 4-2016-0929). Given its retrospective nature, written informed consent was not required by the board to access the clinical data.

### ERCP procedure

The ERCP procedures were performed by six experienced endoscopists who had at least 5 years of experience; each had previously performed > 1,000 ERCP procedures. ERCPs were performed under conscious sedation with propofol and pethidine, and monitoring was done by an anesthesiologist or endoscopist. All ERCPs were conducted under fluoroscopic guidance to diagnose and manage the obstruction using a large (4.2-mm) accessory channel duodenoscope (JF-240, TJF 260V; Olympus Optical Co., Ltd., Tokyo, Japan). Cannulation of the common bile duct (CBD) was attempted with a conventional cannula (Contour ERCP cannula; Boston Scientific, Natick, MA, USA) with or without a guidewire, or with a pull-type sphincterotome (Clever-cut [Olympus Optical] or Autotome RX 44 [Boston Scientific]). A precut papillotomy was attempted when conventional cannulation methods failed.

### Definition

Acute pancreatitis was defined by fulfilment of at least two of the following three criteria: 1) pain in the upper abdomen, 2) serum amylase or lipase concentration more than three times the upper limit of normal, and 3) imaging features of acute pancreatitis on computed tomography (CT) or magnetic resonance imaging [8]. Biliary pancreatitis is related to gallstones/sludge within the gallbladder or bile duct. Biliary pancreatitis was defined by fulfilment at least one of the following criteria: 1) gallstones or biliary sludge on imaging, 2) dilated CBD on imaging (defined as > 8 mm in patients aged ≤ 75 years and > 10 mm in patients aged > 75 years), and 3) alanine aminotransferase more than two times the upper limit of normal [9,10]. A biliary obstruction without cholangitis was defined as a biliary obstruction sign, such as a dilated CBD or jaundice, without any of the symptoms of cholangitis mentioned above. The total length of the hospital stay was defined as the time between admission to and discharge, and the duration of hospitalization after ERCP was defined as the time between performing ERCP and discharge. ERCP-related complications were defined as follows: 1) clinically relevant bleeding, presence of melena, hematochezia, or hematemesis, in combination with a decrease in hemoglobin of 1.3 mmol/L or the need for a blood transfusion; 2) duodenal perforation, diagnosed on plain radiography or CT scan demonstrating free air in the retroperitoneal space outlining the left kidney and psoas muscle; 3) cardiovascular complications, such as myocardial infarction, cerebrovascular accident, or shock; and 4) post-ERCP pancreatitis diagnosed according to the signs and symptoms of pancreatitis with elevated pancreatic enzymes after ERCP [11].

We rated the patient’s pancreatitis severity using the Bedside Index for Severity in Acute Pancreatitis (BISAP) [12]. The BISAP uses five criteria: blood urea nitrogen > 25 mg/dl, impaired mental status as evidenced by disorientation or disturbance in mental status, presence of systemic inflammatory response syndrome (SIRS), age > 60 years, and pleural effusion. SIRS was defined by fulfilment of two or more of the following criteria: pulse > 90 beats/min, > 20 breaths per min, PaCO_2_ < 32 mmHg, temperature > 38°C or < 36°C, and white blood cell count > 12,000 or < 4,000 cells per mm^3^.

### Outcomes

In both groups, the primary outcomes were total length of hospitalization and ERCP-related complications. The secondary outcomes included mortality, technical success rate, and clinical success rate. Technical success was defined as successful removal of stones or sludge from the bile duct. Clinical success was defined as normalization of the serum levels of pancreatic enzymes and relief of symptoms, such as abdominal pain.

### Statistical analysis

All data are expressed as medians (± standard deviation) or n (%), as appropriate. The Mann–Whitney *U* test was used to compare continuous variables, and the chi-square test or Fisher’s exact test was used for categorical variables. All statistical analyses were conducted with SPSS software (ver. 23.0; SPSS Inc., Chicago, IL, USA), and a *P*-value < 0.05 was considered significant.

## Results

### Patient characteristics

**Fig 1:**
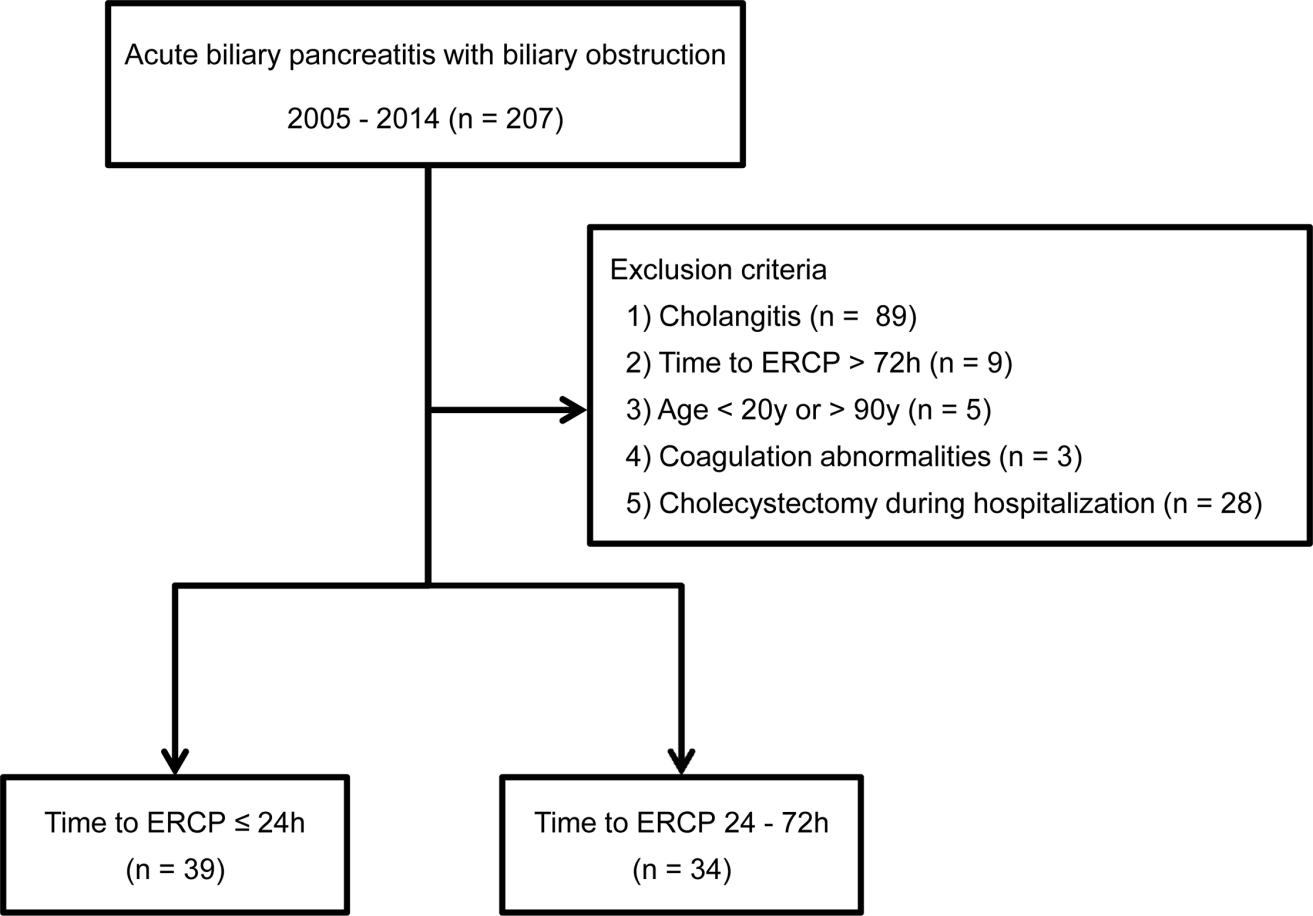
Study population enrolled in the present study. Among 505 patients with acute pancreatitis, 207 patients had a diagnosis of acute biliary pancreatitis. According to the exclusion criteria, a total of 73 patients were enrolled in the present study.

A total of 505 patients diagnosed with acute pancreatitis were evaluated. Among the 505 patients, 207 were diagnosed with ABP and a biliary obstruction. A total of 134 patients were excluded according to the exclusion criteria (89 patients with cholangitis, 9 with time to ERCP > 72 h after admission, 5 aged < 20 or > 90 years, 3 with coagulation abnormalities, and 28 with simultaneous cholecystectomy during hospitalization). Thus, 73 patients were finally enrolled in the present study (Fig 1). The clinical characteristics of the patients are listed in Table 1. Among the patients, 39 (53.4%) underwent urgent ERCP and 34 (46.6%) underwent early ERCP. ERCP duration (mean ± SD) was 13 ± 9 h and 48 ± 12 h in the urgent ERCP and early ERCP groups, respectively. The mean age of the patients was 55.0 and 65.5 years, and the proportion of male patients was 53.8% and 58.8% in the urgent and early ERCP groups, respectively. The level of total bilirubin was higher in the urgent ERCP group than in the elective early ERCP group, and the level of C-reactive protein was higher in the early ERCP group than in the urgent ERCP group. No differences in any other baseline characteristics were observed between the groups.

**Table 1:** Baseline characteristics.

	Variable time to ERCP (n = 73)		*P*-value
	≤ 24 h (n = 39)	24–72 h (n = 34)	
Male	21 (53.8)	20 (58.8)	0.669
Age, years (range)	55.0 (31–90)	65.5 (26–85)	0.089
Symptom on admission*			0.633
Abdominal pain	35 (89.7)	28 (82.4)	
Nausea/vomiting	3 (7.7)	4 (11.8)	
Jaundice	1 (2.6)	2 (5.8)	
Total bilirubin, mg/dL	4.1 ± 4.3	2.3 ± 1.9	0.031
Direct bilirubin, mg/dL	2.9 ± 3.7	2.1 ± 1.4	0.338
ALP, IU/L	167 ± 98	164 ± 135	0.918
r-GT, IU/L	404 ± 337	482 ± 469	0.468
AST, IU/L	218 ± 223	245 ± 235	0.622
ALT, IU/L	288 ± 261	209 ± 166	0.141
Amylase, U/L	1601 ± 1412	1434 ± 1056	0.579
Lipase, U/L	3778 ± 3956	3316 ± 3283	0.596
WBC, /μL	9144 ± 2792	10,318 ± 3768	0.132
CRP, mg/L	6.1 ± 5.9	24.5 ± 30.4	0.011
Diagnosis of biliary pancreatitis			0.124
Elevated total bilirubin	0 (0)	2 (5.9)	
CBD stone	16 (41)	18 (52.9)	
GB stone	6 (15.4)	1 (2.9)	
Bile duct dilatation	0 (0)	1 (2.9)	
Combined	17 (43.6)	12 (35.3)	
Severity index of pancreatitis^†^			0.527
<2	29 (74.4)	23 (67.6)	
≥2	10 (25.6)	11 (32.4)	

Variables are expressed as mean ± SD or n (%).

ALP, alkaline phosphatase; r-GT, γ-glutamyltransferase; AST, aspartate aminotransferase; ALT, alanine aminotransferase; WBC, white blood cell; CRP, C-reactive protein; CBD, common bile duct; GB, gallbladder

*Some patients had more than one symptoms and signs

†BISAP, bedside index for severity in acute pancrieatitis

### Outcomes

The characteristics of ERCP performed in 73 patients are described in Table 2. The technical success rate was 97.4% (38/73) in the urgent ERCP group and 94.1% (32/73) in the early elective ERCP group. However, no difference in technical success rate was detected between the two groups (*P* = 0.476). In 45 (61.6%) patients, bile duct stones were detected and the stones were successfully extracted in 42 (93.3%) of these patients without complications. The reasons for technical failure of ERCP in both groups were mucosal edema or impacted stones, or anatomical difficulties during cannulation, for example due to a diverticulum.

**Table 2:** Characteristics of ERCP procedures performed in patients.

ERCP characteristics	Variable time to ERCP (n = 73)		*P*-value
	≤ 24 h (n = 39)	24–72 h (n = 34)	
Technical success rate	38 (97.4)	32 (94.1)	0.476
Sludge in CBD	7 (17.9)	4 (11.8)	0.461
Stones in CBD	23 (59.0)	22 (64.7)	0.615

Variables are expressed as the n (%).

ERCP, endoscopic retrograde cholangiopancreatography; CBD, common bile duct; P-duct, pancreatic duct.

Table 3 shows the hospitalization duration, ERCP-related complications, pancreatitis-related complications, and severity of pancreatitis of the patients. Diffuse pancreatic swelling suggestive of acute pancreatitis was noted on the initial abdominal CT scan in all patients. However, pancreatic necrosis or pancreatic pseudocysts was not noted on the initial abdominal CT scan. The urgent ERCP group tended to have a longer duration of hospitalization after ERCP compared with the elective early ERCP group (5.1 vs. 3.4 days, *P* = 0.085). The total length of hospital stay, which may be associated with cost, was not significantly different between the urgent ERCP group and the elective early ERCP group (5.9 vs. 5.7 days, *P* = 0.174).

**Table 3:** Hospitalization day and complications.

	Variable time to ERCP			*P*-value
	≤ 24 h (n = 39)		24–72 h (n = 34)	
Total length of hospital stay, days*	5.9 ± 5.0		5.7 ± 2.5	0.174
Duration of hospitalization after ERCP, days**	5.1 ± 5.0		3.4 ± 2.6	0.085
Post-ERCP complications				0.113
Sepsis	1 (2.6)		0 (0)	
Cholangitis	3 (7.7)		1 (2.9)	
Bleeding	2 (5.1)		0 (0)	
Perforation	0 (0)		0 (0)	
Complications due to pancreatitis^†^	1 (2.6)		2 (5.8)	0.476
Severity of pancreatitis^††^	0.9 ± 0.9		1.1 ± 0.9	0.670

Variables are expressed as the mean ± SD or n (%).

*The total length of hospital stay is the duration from admission to discharge.

**The duration of hospitalization after ERCP is the duration from ERCP to discharge.

†All pancreatitis-related complications are renal failure

††BISAP, bedside index for severity in acute pancreatitis

Seven patients experienced ERCP-related complications. No significant difference was observed in the complications rate between the two groups (*P* = 0.113). Two patients in the urgent ERCP group bled after sphincterotomy. All bleeding events were controlled by a local injection of epinephrine (dilution, 1:10,000) and closed observation; no blood transfusions were needed. No other immediate complications, such as perforation of the duodenal wall, were detected in either group.

We also investigated pancreatitis-related complications, which were defined according to the Atlanta classification [13]. The only systemic complication was renal failure, seen in three patients. Renal failure was defined as a serum creatinine level > 2 mg/dl after rehydration or a need for hemofiltration or hemodialysis. No significant difference was detected between the urgent ERCP group (< 24 h) and early ERCP group (24–72 h) regarding the frequency of pancreatitis-related complications (2.6% vs. 5.8%, *P* = 0.476) (Table 3).

We chose the BISAP severity scoring system to measure pancreatitis severity. The proportions of patients according to BISAP scores were as follows: 0 (n = 27, 37%), 1 (n = 25, 34.2%), 2 (n = 16, 21.9%), and ≥ 3 (n = 5, 6.8%) (Table 4). No significant difference in median pancreatitis severity score was observed between the groups (*P* = 0.670) (Table 3).

**Table 4:** Number of patients stratified by the BISAP point score.

BISAP score^†^	Variable time to ERCP		Total
	≤ 24 h (n = 39)	24–72 h (n = 34)	
0	18 (46.2)	9 (26.5)	27 (37.0)
1	11 (28.2)	14 (41.2)	25 (34.2)
2	8 (20.5)	8 (23.5)	16 (21.9)
≥3	2 (5.1)	3 (8.8)	5 (6.8)

Variables are expressed as n (%)

†BISAP, bedside index for severity in acute pancreatitis

## Discussion

In the present study, we investigated the need for urgent ERCP within 24 h to control biliary obstructions in patients with ABP. Based on the results of this study, urgent ERCP is not superior to elective early ERCP in terms of complications or hospitalization duration. Furthermore, there were no differences in the technical or clinical success rates between the two groups.

Several clinical trials have aimed to identify the proper timing of ERCP for patients with ABP, to reduce the rate of mortality and complications. Neoptolemos et al. [14] showed that patients with predicted severe acute pancreatitis had fewer complications if they underwent early ERCP (within 72 h of admission; 24% vs. 61%, *P* < 0.05). On the other hand, Folsch et al. [15] reported that early ERCP was not beneficial in patients with ABP but without obstructive jaundice and cholangitis. A recently published meta-analysis showed no significant difference in mortality rate according to the timing of ERCP (< 24 h vs. < 72 h) [6].

However, the timing of the procedure was defined differently in previous studies including patients with ABP, such as < 24 h after admission [16,17], < 72 h after admission [7,14,18], and < 72 h after symptom onset [15]. In the present study, the patients were divided into two groups according to the time of intervention after admission, which has more practical applicability to daily practice. In previous studies, the diagnostic criteria for gallstone pancreatitis and cholangitis also varied [2,5,19]. One reasons for this is that it is occasionally difficult to diagnose patients with CBD stones or gallstone pancreatitis. Moreover, commonly used biochemical and radiological predictors of biliary obstruction are unreliable during the early phase of ABP. The liver function test is normal in about 15–20% of patients with ABP, although it can be commonly checked, and abdominal ultrasonography has a low sensitivity (27–50%) for CBD stones or dilatation [9,20]. In the present study, the diagnosis of biliary pancreatitis was based on imaging and laboratory results according to international consensus guidelines [2,5].

Previous animal and human studies have suggested that the severity of pancreatitis is related to the duration of a bile duct obstruction, which is therefore a critical factor contributing to the severity of pancreatitis [21,22]. Thus, early ERCP within 72 h may be helpful for decompressing the biliary obstruction, although our study demonstrated that urgent biliary intervention within 24 h would not lead to the additive benefit of reduced total hospital stay and complication rates. In particular, the cost-effectiveness of urgent ERCP should also be considered [23].

In previous studies, the cut-off level of total bilirubin for defining cholestasis varied and patients with total bilirubin levels of 1.2–5.0 mg/dL were included in the cholestatic group [6,24,25]. In this study, the level of total bilirubin was higher in the urgent ERCP group than in the elective early ERCP group, which might have affected the timing of ERCP in patients with cholestasis. We performed a subgroup analysis to exclude the effect of total bilirubin on total length of hospital stay and complications. The subgroup analysis after matching the level of total bilirubin still did not show a significant difference in total length of hospital stay or complications between the groups (S2 Table).

We performed pre-specified subgroup analyses according to the predicted severity of pancreatitis. No significant differences were detected in the risk of complications or total length of hospital stay between two groups, regardless of predicted severity (Table 5). In recent clinical trials and guidelines, ERCP is not recommended in most patients with ABP for whom there is a lack of evidence of biliary obstruction or cholangitis, regardless of predicted severity [2,4,5]. In this study, we found no clinical difference between the two groups (<24 h vs. 24–72 h) in terms of ERCP-related complications or total length of hospital stay, regardless of predicted severity.

**Table 5:** Hospitalization day and complications stratified by BISAP score†.

		Variable time to ERCP		*P*-value
		≤ 24 h (n = 39)	24–72 h (n = 34)	
BISAP < 2	Total length of hospital stay, days*	4.5 ± 2.3	5.6 ± 1.8	0.065
	Duration of hospitalization after ERCP, days**	3.6 ± 2.3	2.9 ± 1.7	0.228
	Post-ERCP complications	4/29	0/23	0.063
	Complications due to pancreatitis	0/29	0/23	NA
BISAP ≥ 2	Total length of hospital stay, days	10.1 ± 7.6	6.5 ± 3.4	0.184
	Duration of hospitalization after ERCP, days	9.3 ± 7.5	4.3 ± 3.6	0.071
	Post-ERCP complications	2/10	1/11	0.475
	Complications due to pancreatitis	1/10	2/11	0.592

Variables are expressed as the mean ± SD or n (%).

*The total length of hospital stay is the duration from admission to discharge.

**The duration of hospitalization after ERCP is the duration from ERCP to discharge.

†BISAP, bedside index for severity in acute pancreatitis.

NA, not applicable.

This study had several limitations. First, it used a nonrandomized, retrospective design. The optimal study design is a prospective, randomized control trial, not an observational study. However, we tried to exclude confounders related to ERCP timing, such as cholangitis, operation, and age, to reduce selection bias. Second, the present study had a small sample size. Table 3 shows that there were complication rates of and 1/34 in the urgent and early ERCP group, respectively. The number of patients with complications was lower in the early ERCP group than in the urgent ERCP group. Even if the proportion of complications in the two groups did not differ significantly, there was a possibility of a type II error considering the small sample size. Because of the small number of patients per group, the study was underpowered to detect meaningful differences. It might indeed be that early ERCP is associated with a lower complication rate compared to urgent ERCP.

This is the first study to evaluate the optimal timing of ERCP for patients with ABP and a biliary obstruction without cholangitis. We found no clinical difference between the two groups (< 24 h vs. 24–72 h) in terms of ERCP-related complications or total hospitalization duration. In conclusion, urgent ERCP is not superior to early ERCP in patients with biliary pancreatitis without cholangitis.

## Supporting information

S1 Table Hospitalization day and complications including patients with cholecystectomy. Cholecystectomy during the index admission is recommended strategy for managing patients who were diagnosed biliary pancreatitis and gallstone. Further, we added those patients with cholecystectomy (n = 28) and analyzed hospitalization day and complications according to variable time to ERCP.
(PDF)

S2 Table Hospitalization day and complications after matching the level of total bilirubin. (PDF)

## Abbreviations

ERCP: Endoscopic retrograde cholangiopancreatography
ABP: Acute biliary pancreatitis
CBD: Common bile duct
CT: Computed tomography
