# Supplementary material for: Urgent endoscopic retrograde cholangiopancreatography is not superior to early ERCP in acute biliary pancreatitis with biliary obstruction without cholangitis
Source: PLoS One. 2018 Feb 5;13(2):e0190835. doi: 10.1371/journal.pone.0190835 (PMC5798765; doi:10.1371/journal.pone.0190835)
Supplement: S1 Table — Cholecystectomy during the index admission is recommended strategy for managing patients who were diagnosed biliary pancreatitis and gallstone. Further, we added those patients with cholecystectomy (n = 28) and analyzed hospitalization day and complications according to variable time to ERCP. (PDF) [file pone.0190835.s001.pdf]

S1 Table. Hospitalization day and complications including patients with cholecystectomy. Cholecystectomy during the index admission is recommended strategy for managing patients who were diagnosed biliary pancreatitis and gallstone. Further, we added those patients with cholecystectomy (n = 28) and analyzed hospitalization day and complications according to variable time to ERCP.

	Variable time to ERCP		*P*-value
	≤ 24 h (n = 54)	24–72 h (n = 47)	
Total length of hospital stay, days*	6.8 ± 5.0	8.2 ± 5.6	0.192
Post-ERCP complications			0.122
Sepsis	1 (1.9)	0 (0)	
Cholangitis	3 (5.6)	1 (2.1)	
Bleeding	2 (3.7)	0 (0)	
Complications due to pancreatitis†	2 (3.7)	3 (6.4)	0.536
Severity of pancreatitis††	0.7 ± 0.8	1.0 ± 0.9	0.168

Variables are expressed as the mean ± SD or n (%).

*The total length of hospital stay is the duration from admission to discharge.

†All pancreatitis-related complications are renal failure.

††BISAP, bedside index for severity in acute pancreatitis.
