# Supplementary material for: Urgent endoscopic retrograde cholangiopancreatography is not superior to early ERCP in acute biliary pancreatitis with biliary obstruction without cholangitis
Source: PLoS One. 2018 Feb 5;13(2):e0190835. doi: 10.1371/journal.pone.0190835 (PMC5798765; doi:10.1371/journal.pone.0190835)
Supplement: S2 Table — (PDF) [file pone.0190835.s002.pdf]

S2 Table. Hospitalization day and complications after matching the level of total bilirubin

	Variable time to ERCP		*P*-value
	≤ 24 h (n = 14)	24–72 h (n = 14)	
Total bilirubin	2.7 ± 1.6		
Total length of hospital stay, days*	4.8 ± 2.8	6.9 ± 3.5	0.084
Duration of hospitalization after ERCP, days†	4.1 ± 2.8	3.2 ± 2.5	0.403
Post-ERCP complications			0.541
Cholangitis	2 (14.3)	1 (7.1)	

Variables are expressed as the mean ± SD or n (%).

*The total length of hospital stay is the duration from admission to discharge.

†The duration of hospitalization after ERCP is the duration from ERCP to discharge.
